# An Improved Method for the Detection and Quantitative Determination of Arsenic in Cases of Poisoning

**Published:** 1844-10-19

**Authors:** 


*An Improved Method for the Detection and Quantitative Determination of Arsenic in Cases of Poisoning.* By Drs. Fresenius and von Babo.

The authors, after detailing the objections, to the means at present adopted for detecting arsenic, as that of Reinsch, Marsh, and the various modifications which have been proposed of this latter method, give the process which forms the subject of this communication. Part of the material to be tested is treated with hydrochloric acid, and chlorate of potash added, assisted by heat, and when sufficiently acted upon, filtered and the solution concentrated; solution of sulphurous acid is next added in excess, and this excess afterwards expelled by heat. Sulphuretted hydrogen is then passed through the solution to saturation, ammonia added, and the whole lightly covered and kept warm until the odour of the gas has disappeared. The precipitate is collected on a filter, and is next acted upon by fuming nitric acid, added by degrees until the whole is moistened, and is then reduced to dryness in the water bath. It is next moistened with hydrated sulphuric acid, and heated in a water bath for two or three hours, and finally to about 302°, Fahr. The dried and charred residue thus obtained is treated with from ten to twenty parts of distilled water, hydrochloric acid added, and again precipitated as sulphuret, collected, and ammonia added, and the ammoniacal solution evaporated to dryness, and dried at 212°; the charred residue, and anything that remains undissolved by the ammonia, must be tested for lead, mercury, bismuth, copper, &c. The reduction of the resulting sulphuret of arsenic is then fully described, and the apparatus into which it is to be conducted figured. The process consists in mixing the sulphuret with dry carbonate of soda and caynide of potassium, and introducing it into the reduction-tube, dry carbonic acid being passed over it, while it is gently warmed, so as to expel all moisture; the flame of a spirit-lamp is applied to the tube beyond the materials, for the purpose of decomposing the liberated vapours, and another strong flame is gradually applied to the mixture until all arsenic is expelled. The reduced arsenic forms a film in advance of the first spirit-lamp flame; should zinc or antimony be present, they will be obtained in their metallic state by dissolving in water the residue found in the reduction tube.—*London Med. Times.*

